# Navigating trust and health in India: the influence of social status and neighbourhood environment

**DOI:** 10.1186/s12889-024-19826-7

**Published:** 2024-10-01

**Authors:** Shrestha Saha

**Affiliations:** Nanyang Technological University, Singapore, Singapore

**Keywords:** Generalised trust, Particularised trust, Health, India, Multilevel modelling, Social status, Income inequality

## Abstract

**Background:**

The research on the relationship between interpersonal trust and health has primarily focused on Western contexts, with scarce attention in developing contexts. Addressing this gap, the study examines the association between interpersonal trust (both generalised and particularised) and health outcomes (self-rated health /SRH, and depression) among Indian adults, considering the moderating roles of social statuses (gender and caste) and macro-level factors like district-level income inequality.

**Methods:**

The study draws on data from the World Health Organization's (WHO) Study on global AGEing and adult health (SAGE) Wave-1, collected between 2007 and 2010. This dataset provides a comprehensive overview of health outcomes, including self-rated health (SRH) and depression, socio-cultural status of adults aged 18 and above in India. Additionally, district-level data on income inequality, quantified through the Gini index, were incorporated to examine the influence of contextual socioeconomic influence on the trust-health relationship. Multilevel regression analysis with interaction effects with social statuses and income inequality at district was employed in the analysis to investigate the intricate relationship between interpersonal trust (both generalised and particularised) and health outcomes.

**Results:**

The study reveals that while generalised trust does not directly influence depression or SRH, particularised trust acts as a protective factor for both health outcomes. Gender-specific interaction effect shows that generalised trust reduces depression among males and improves SRH among females. Notably, caste does not significantly moderate the trust-health relationship. High district-level income inequality, however, modifies these associations: generalised trust is associated with improved SRH in areas of high inequality, whereas particularised trust correlates with increased depression in these districts.

**Conclusion:**

The findings highlight the complex dynamics between interpersonal trust, social status, and income inequality in shaping health outcomes in India. Generalised trust emerges as a potential buffer against the health-detrimental effects of income inequality, providing crucial insights for developing targeted health interventions. These results offer valuable guidance for global health policymakers and practitioners in effectively allocating development aid to enhance health outcomes, especially among the most marginalised groups.

**Supplementary Information:**

The online version contains supplementary material available at 10.1186/s12889-024-19826-7.

## Background

The role of interpersonal trust in health and wellness has been a subject of expanding interest across various fields of social science [63], public health [33] and epidemiology [36]. Interpersonal trust is considered 'moral resource' that promotes mutual reciprocity within social networks and exerts a defensive impact on individuals' health and well-being. Trust might influence health through some possible mechanisms: providing social support, enforcing informal social control, production of collective efficacy, and the dispersion of health-related knowledge [7, 35]. Conceptually, interpersonal trust envelops a wide range of thoughts and ideas. Fukuyama ([23], p. 26) defined interpersonal trust as "the expectation that arises within a community of regular, honest and cooperative behaviour based on commonly shared norms on the part of other members of that society". Social psychologists consider trust a 'core personality trait', mainly symptomatic of optimism and the ability to control the world or at least one's own life [66]. Delhey and Newton ([16], p.95) defined trust as a 'part of a broader syndrome of personality characteristics that includes optimism, a belief in cooperation, and confidence that individuals can resolve their differences and live a satisfactory social life together".

Previous theoretical studies have identified two distinct types of interpersonal trust, generalised and particularised, and highlighted their distinct role in individuals' lives [72]. Generalised trust refers to a disposition to trust people in general, including strangers and individuals beyond one's immediate social circle. It allows individuals to trust and rely on others beyond their immediate social network and can facilitate access to resources and support. It has been found to contribute to a sense of control, provide health-related information, and promote healthy behaviours. On the other hand, particularised trust is developed within close social relationships, such as family, friends, neighbours, and colleagues, based on regular interactions and familiarity [19]. Particularised trust involves 'in-group homogeneity' where trust within a closely-knit group ensures that resources are selectively distributed among its members, differing fundamentally from generalised trust. Social support at the interpersonal level provides the most convincing link to explain the relationship between particularised trust and health. This form of trust provides a guarantee that support from a trusted friend or relative is available when needed, influencing health through the encouragement of support-seeking behaviors [19]. Trust and social support, while related, serve different functions in social interactions. Trust facilitates the seeking and receiving of support from both broader communities and individual relationships, influencing the type and efficacy of the support received.

Due to such distinction, the scope of generalised and particularised trust differs, and their health-protective mechanisms are often exclusive to each other. Most of the research conducted in the Western context treated interpersonal trust as a monolithic unidimensional concept, typically measured as respondents' agreement that 'most people can be trusted' and thus overlook particularised trust or the trust in specific individuals like family, friends, and significant other. Due to the widespread emphasis on generalised trust, little is known about particularised trust and its influences on health. While many scholars have begun to highlight the theoretical differences between these two types of trusts, only a handful of studies have simultaneously examined the role of two widely discussed dimensions of interpersonal trust that are generalised and particularised [25, 37]. This study aims to distinguish generalised and particularised trust, concentrating on their possible impact on health.

## Research gap and objectives

Numerous examples (predominantly in developed countries) in different periods have established that interpersonal trust is significantly associated with physical, mental and self-rated health status [55, 71]. Interpersonal trust significantly influences health, with generalised and particularised trust having distinct impacts. Generalised trust enhances individual and community health by reducing social isolation, boosting security, fostering social interactions, and building confidence. Generalised trust doesn’t provide emotional and instrumental support as one cannot seek help from strangers, rather, it improves health by enhancing social integration, facilitating collective efficacy and mutual reciprocity and enforcing health-protective social norms. This type of trust helps communities manage adversities like unemployment or illness by facilitating shared resources and mutual support [13, 28, 39, 73]. Particularised trust, grounded in close social ties, is recognised for its psychological benefits, offering substantial emotional and instrumental support that acts as a buffer against distress and chronic illness [25, 34, 44, 57]. This type of support, encompassing everything from practical help to emotional care, is pivotal in reducing chronic stress and boosting mental health and well-being.

Despite these findings, the applicability in less-developed, non-Western contexts remains uncertain. Among a handful of research that has attempted to examine this contextual relevance, the established hypotheses have been generally found to be violated, particularly in developing countries (for example, Ghana [3]) and least-developed countries (for example, sub-Saharan Africa [2]). In Ghana, increased generalised trust was associated with higher levels of depression among adults. In sub-Saharan Africa, generalised trust was associated with increased depressive symptoms in low-trust districts. In other words, people who trust generalised others in a low generalised trust society might get exploited. The differences are not strictly based on the level of economic development. This is particularly evident in a study conducted in Chile, which is a developing country, but an inverse relationship between individual-level trust to mental was exhibited, which is similar to what is found in high-income western countries [54]. Such a mixed bag of evidence implies a critical need to reassess and expand the predominantly Western-centric literature to incorporate diverse global contexts.

In India, limited research has explored the impact of interpersonal trust on the health and well-being of the elderly, with studies by Samanta [56] and Himanshu et al. [30] indicating that higher trust levels can significantly reduce psychological distress among older adults. Himanshu et al. [30] specifically notes that increased trust helps alleviate psychological distress, while Samanta [56] suggests that fostering community trust may be more crucial for elderly well-being than merely improving living arrangement. These findings underscore the potential benefits of further research in this area, which could lead to significant improvements in the health and well-being of the elderly in India.

Unlike more affluent societies with robust social safety nets, many in India rely on direct social networks for health-related decisions due to sparse social protections and significant geographical and infrastructural challenges [11, 53]. These networks, pivotal in managing health care decisions and providing care, emphasize the critical role of interpersonal trust in navigating health management within resource-constrained environments.

This research addresses the influence of interpersonal trust on health outcomes within India's distinct socioeconomic and cultural contexts, examining how ascriptive social statuses and broader contextual features moderate the relationship between trust and health. Caste and gender represent critical ascriptive social statuses in India. These statuses not only signify fundamental social divisions but also serve as pivotal starting points in the life course and central markers of social identity. Thus significantly impacting individuals' psychosocial attributes and interaction with society. While extensive research outside India has highlighted how the subordination of women and ethnic minorities impacts network inequality and trust development across both developed and developing nations, such focused analyses remain limited within the Indian setting. This research aims to bridge this gap by investigating how variations among different caste and gender groups moderate the relationship between health and interpersonal trust, marking a new avenue of exploration within the field.

At the contextual level, the study explores how the distribution of resources, varying significantly across different regions, affects trust and, subsequently, health outcomes. Utilizing a district-level income inequality index, it assesses the role of economic disparities in shaping the dynamics of trust and health. Previous research has shown that societal inequality undermines social relationships, promotes mistrust, and leads to lower levels of generalized trust, adversely affecting health outcomes. In contrast, areas with fair resource distribution and low corruption typically show higher interpersonal trust and better health outcomes. This analysis aims to reveal how regional resource allocation variations affect interpersonal trust and health in India's diverse socioeconomic landscape.

To explore the trust-health nexus, this study leverages nationally representative data from 11,230 participants across 136 districts in six states, each representing diverse geographical regions and socioeconomic stages in India. It specifically examines the impact of interpersonal trust on self-rated health (SRH) and depression. Interpersonal trust significantly influences these health outcomes by enhancing social interactions and strengthening community bonds. Notably, high levels of community trust reduce mental distress, particularly for those who are socially isolated, by fostering feelings of security and belonging—crucial elements for mental health and perceived health status. These factors are especially critical in influencing self-rated health and depression, as they directly shape individuals' perceptions of their well-being and actual mental health status. The insights provided are expected to inform policymakers in India and similar environments, deepening how trust, social stratification, and health are intricately connected.

## Interpersonal trust and health: role of moderating factors

### Role of social status

Interpersonal trust, as an individual's network resource, is unevenly distributed across social groups, and this unequal access extends to the differential returns accrued from interpersonal trust [41]. The existing research, primarily from Western contexts, highlights significant disparities in the distribution of interpersonal trust, with general access to and utilization of generalized trust typically being lower for women and racial minorities. This differential access to types of trust can lead to distinct health outcomes. While particularized trust provides strong support within tight-knit communities, the limited access to generalized trust restricts broader societal integration and access to diverse resources, which are crucial for health and well-being [20, 27, 42, 61]. Research shows that while women and racial minorities often enjoy higher levels of particularized trust within homogeneous networks, their access to generalized trust is typically lower, limiting their integration into broader society and access to diverse health resources [20, 27, 42, 61]. Consequently, despite the strong support from particularized trust, the reduced access to generalized trust among women and racial minorities can exacerbate health disparities by limiting opportunities and resources available through wider, more diverse network.

Furthermore, the psychosocial risks associated with poor health are heightened among disadvantaged groups by factors such as social isolation, discrimination, and marginalization, which foster mistrust and pessimism. In such closely-knit networks, the demands of emotional labor on women and racial minorities can exacerbate psychological stress, particularly when generalized trust is lacking, making it harder to access broader support networks [24, 38]. Consequently, while particularized trust provides substantial in-group support, the lack of generalized trust hinders broader interactions and access to varied resources, deepening health inequalities and underscoring the complex relationship between trust and health in marginalized communities.

### Role of income inequality

The link between interpersonal trust and health is significantly shaped by socio-cultural contexts, with income inequality being a crucial factor in developed nations. Research indicates that high-income inequality deteriorates social relations and generalized trust, negatively affecting health outcomes. Seminal studies, such as Wilkinson [67], found higher income inequality with lower life expectancy in affluent countries due to increased social strife and reduced trust, which elevates mortality rates. Further, Kawachi et al. [36] demonstrated how in the U.S., trust mediates the relationship between income inequality and mortality, highlighting the complex interaction between economic conditions and social trust.

Conversely, Islam et al. [31] suggest that in settings with marked income disparities, social trust might buffer the adverse effects of economic inequality by bolstering network-mediated support. This role of social capital is critical in less egalitarian societies where healthcare access is patchy, making trust a vital community resource.

Additionally, there is often a heavier reliance on particularised trust in areas with significant income inequality, corruption, and high crime rates. This type of trust is crucial for managing financial and health crises through support from close-knit groups like neighbours and friends [12, 63]. Thus, in these environments, limited resources force a dependence on intimate, trust-based relationships to uphold social cohesion and navigate challenges.

## Hypotheses

### Micro-level factors

#### Social status: gender and caste

Caste hierarchy and gender hierarchy are fundamental pillars of traditional Indian social structure, and they continue to be the dominant sources of socioeconomic inequality, health disparities, and equality of opportunity for upward social mobility.

Gender is considered a symbol of inequality and disadvantage in India; research in India has highlighted the ways in which gender inequality and disadvantage intersect with biological, social, and cultural factors that impact women's health [15, 18]. Lower socioeconomic status, heavier reproductive roles, and gender-specific socialisation render women vulnerable to health issues. Persistent gender roles in family and work compound these challenges, disadvantaging women [40, 52].

Existing research conducted in non-Indian contexts has highlighted the influence of gender stereotypes on networking opportunities. Traditional roles, limited public interaction, and gendered work expectations hinder broader social connections create challenges for women in building social connections beyond their immediate social circles. Consequently, women may experience lower levels of trust, resulting in a lack of social support that can adversely affect their health and well-being [14, 43]. Given India's deep gender disparities, considering gender is pivotal in understanding trust's health link.

The caste system in India provides a profound cultural context for understanding intergroup disparities and the dynamics of social relations between groups [10, 58]. Originating from the ancient 'Varna' system, caste categorizes society into four endogamous groups based on traditional occupations, with each group inheriting distinct levels of power and prestige [8]. Like race in the United States, caste is a birth-ascriptive social status that significantly influences life opportunities by affecting access to network resources and social capital.

The most socially and economically disadvantaged groups, notably the Scheduled Castes (SCs), have historically faced extensive discrimination and oppression from upper caste groups across various spheres of life, including education, employment, and the judicial system. Despite changes in the societal perception and impact of caste over time, it remains a critical driver of disparities, fostering social and political tension as well as inter-community distrust due to ongoing caste-based discrimination, marginalization, and alienation [6, 49].

This backdrop of historical and ongoing exclusion profoundly impacts interpersonal trust within and between caste groups. The entrenched distrust and lack of mutual reciprocity among different caste groups exacerbate social stress and anxiety, which in turn negatively affect both physical and mental health outcomes [68, 69]. Health disparities are particularly pronounced among the Scheduled Castes, who consistently exhibit poorer health indicators compared to middle and upper castes, a consequence of substandard living conditions, constrained social mobility, and discrimination within healthcare settings [1, 9, 50].

Given these dynamics, this paper explores the impact of caste on interpersonal trust and its subsequent effects on health. It posits those differences in trust—both generalized and particularized—among caste groups can significantly influence health outcomes. This leads to the formulation of specific hypotheses:

- **H1: **Men will have higher health benefits from generalised and particularised trust than women.
- **H2: **Scheduled caste members will have lower health benefits from the generalised and particularised trust than non-scheduled caste members.

### Macro-level factor

#### District level income inequality

Despite experiencing significant economic growth, India has witnessed a rise in economic inequality at the national level and within and between states [5, 59]. Such differences are partly due to the growing divergences of income and non-inclusive economic development within the country that has existed since independence [29]. India provides an excellent case study due to its distinct extremes and also for the fact that the distance between the richest and poorest states has increased substantially over the post-independence period [5]. The Indian context exemplifies significant disparities and highlights the contrasting fortunes of states with evidence of divergence, polarisation, and the formation of distinct economic clubs. While some northern and western states (Haryana, Maharashtra, Punjab, and Gujarat) have enjoyed sustained prosperity, several southern states (such as Karnataka, Kerala, and Tamil Nadu) have experienced notable economic growth. However, a disconcerting pattern of persistent poverty remains in certain states like Assam, Bihar, Odisha, Madhya Pradesh, and Rajasthan over an extended period [5]. Further, Intra-state inequality indices tend to be higher in districts with higher levels of living and development than in districts with lower levels of development [47]. Analyses of the role of inequality in public health tend to be lower in the poorest countries, specially at the subnational level. This research sheds light on the importance of interpersonal trust in the context of income disparities at districts and its potential impact on the health of adults. In the context of India, districts stand as the most basic administrative entities where elected district councils formulate plans for infrastructure, development, and various essential services [48]. District-level analysis can play a crucial role in guiding decentralised planning and ensuring the success of health intervention programs aimed at reducing inequities in the country. In high-income inequality districts, individuals may experience negative emotions such as distrust, shame, and exclusion due to their heightened awareness of their comparative socioeconomic standing and relative isolation from the rest of the population. These negative emotions may lead to chronic stress and subsequently affect their health negatively. Therefore, the study proposes.

- **H3a.** The health benefit of interpersonal trust (generalised and particularised trust) on health status SRH and depression will be reduced in districts with a higher level of income inequality.

On the other hand, in highly unequal districts, people may be compelled to rely on their social networks and interpersonal trust to access basic medical services or receive adequate care. In such cases, little amount of trust becomes particularly crucial as a "substitute" for the lack of formal infrastructure and health care services. Therefore the study proposes.

- **H3b**. The health benefit of interpersonal trust (generalised trust and particularised trust) on health status SRH and depression will enhance in districts with a higher level of income inequality.

## Data and methods

The study used data from two sources: individual-level data from the WHO Study on global AGEing and adult health (SAGE) wave 1 (2007–10) for India. The SAGE Wave 1 India survey included 11,230 completed interviews from six states (Assam, Karnataka, Maharashtra, Rajasthan, Uttar Pradesh, and West Bengal), with 4,670 interviews with individuals aged 18–49 and 6,560 interviews with individuals aged 50 and over. The study retained all 11,230 respondents as collected in Wave 1. The sample was therefore inclusive of all participants initially surveyed in the six states. Although WHO-SAGE is designed as a longitudinal dataset, this study specifically utilizes data from wave 1 because subsequent waves were not published at the time of the analysis. The Wave 2 data has only been published very recently, and it was inaccessible before the analysis was conducted. The district level (contextual level) estimates of income inequality were taken from the published source [47].

### Variable and measures

#### Self-rated health (SRH)

Most studies of SRH use a categorical approach to differentiate between varying health levels. For this study, respondents were asked, "how would you rate your health today?" with answers recorded on a five-point scale ranging from 1 (very good) to 5 (very bad). I have classified "very good" and "good" as indicative of good health, and "moderate" (equivalent to fair), "bad," and "very bad" as indicative of bad health. This classification follows a conventional approach widely adopted in health research, where typically "excellent," "very good," and "good" are categorized as good health, while "fair," "poor," and "very poor" are deemed bad health [45, 62].

##### Depression

In the WHO-SAGE study, depression was assessed using a combination of self-reported diagnoses and an evaluation of depressive symptoms experienced over the past 12 months. The diagnostic criteria applied were based on the International Classification of Diseases, 10th Edition, Diagnostic Criteria for Research (ICD-10-DCR). Participants were classified as having depression if they reported a clinical diagnosis of depression or if their symptom reports satisfied the ICD-10 criteria. The ICD-10 is a widely recognized and validated medical classification system employed by the World Health Organization for diagnosing and researching diseases and health-related issues.

#### Interpersonal trust

Generalized trust is measured by whether respondents have trust in generalized others in society, assessed with the question: “*Generally speaking, would you say that most people can be trusted or that you can’t be too careful in dealing with people?*” This question implies a broad evaluation of trust towards society at large, reflecting an individual's general inclination to trust people outside their immediate social circles.

Particularized trust is measured by whether respondents have someone they can confide in, evaluated with the question: *“Do you have someone you can trust and confide in?”.*

This question implies a specific evaluation of trust within close, personal relationships, highlighting whether individuals have trusted confidants in their immediate social environment.

In the statistical analysis, both of the trust variables are treated as binary response: 0 = Not having trust, 1 = Having trust.

##### Social statuses

Gender (1 = male) and Caste (1 = Scheduled Caste) are dummy variables in the analysis. The WHO-SAGE data classifies Caste into four categories: "Scheduled Caste," "Scheduled Tribes," "No Caste or Tribes," and "Others." The WHO-SAGE dataset does not include caste data that aligns with the traditional Varna system of India, which divides the population into four hierarchical endogamous groups: Brahmins, Kshatriyas, Vaishyas, and Shudras. Due to the dataset's limitations, no further granularity is available for caste classification. For this study, I simplified the caste groups into a binary classification: Scheduled Caste versus Others. The data indicates that Scheduled Caste and Scheduled Tribe comprise 23% of the sample, while the non-Scheduled Caste group, including "Others" and "No Caste or Tribes," accounts for approximately 76%. The study specifically analysed Scheduled Castes due to their unique socioeconomic challenges and historical disadvantages, which have been well documented in the literature and are relevant to health disparities.

#### Other socioeconomic variables

Among demographic variables, age is measured as a continuous variable. Religion is categorised into two binary groups, Hindu (Hindu = 1) versus Others (others = 0) and Muslim (Muslim = 1) versus Others (others = 0). The categorisation was made based on the distribution frequencies observed within the sample and the context of existing research. In the dataset, Hindus represent approximately 84% of the population, while Muslims account for about 12%. The remainder of the population, constituting 4%, includes various other smaller religious groups. Given the substantial majority of Hindus and the significant representation of Muslims as the second-largest religious group, binary categorisation is opted to ensure analytical clarity and robustness. The primary aim was to capture the contrast in health outcomes between these dominant groups and the combined categories of all other religions. Marital status is also a dummy variable (1 = currently married) place of residence (urban = 1) is also a dummy variable. The respondent’s education level and household wealth quintile, have been taken as indicators of socio-economic status. Educational level is measured by respondents’ highest level of completed education and counted as 0 = ‘no formal education’, 1 = ‘less than primary school’, 2 = ‘Primary school completed’, 3 = ‘Secondary school completed’, 4 = ‘High school (or equivalent) completed’, 5 = ‘College/university completed’, 6 = ‘Post-graduate degree completed’. These categories are further broadly grouped as 0 = ‘less than high school’ and 1 = ‘high school and above’. The second indicator of SES are measured by household wealth quintile to capture the relative inequalities in income at households. A statistical division of sample households into five equal parts, based on wealth (assets). Quintile 1 contains the poorest households, and quintile 5, the richest households. We categorized wealth quintile into two groups 1–3 = ‘low income’ 4–5 = ‘high income’. The final version of household income is 0 = Low income 1 = High income.

##### Income inequality

Income inequality for 136 districts is measured by Gini index. The district-level Gini index were obtained from the published source by Mohanty et al. [47]. They employed consumption-based inequality indices across India's districts by combining data from the 66th and 68th rounds of the National Sample Survey on consumption expenditure, conducted in 2009–2010 and 2011–2012, respectively. Both rounds included Type 1 Schedule, which surveyed consumption over a 30-day reference period for food and over both 30-day and 365-day reference periods for non-food items, and Type 2 Schedule, which surveyed consumption over 7-day and 30-day reference periods for food and 30-day and 365-day reference periods for non-food items. The 66th round (Schedule 1.0) encompassed a sample of 100,794 households across 610 districts in India, while the 68th round covered a sample of 101,651 households in 623 districts. Total number of districts utilised is 623.

## Analytical strategy

### Integration of district-level data

The WHO-SAGE (2007–10) dataset employed a multi-stage cluster sampling design, collecting data from 11,230 individuals across 136 districts in rural and urban settings. While the dataset primarily provides individual-level data, it identifies the district of each respondent, making it possible to link these individuals to broader district-level variables. For the multilevel (two-level) analysis, I introduced a district-level Gini index as a contextual variable. This external data (from [47]) was merged with the SAGE data by associating each respondent with their respective district's Gini index based on the district names provided in the dataset. This integration facilitated a richer analysis by combining individual (level 1) and district-level (level 2) data to more effectively explore the influence of district-level income disparities on trust-health relationships.

### Regression model

This study hypothesised that people’s health outcomes are influenced not only by individual-level factors but also by the characteristics of the broader social context (here districts) in which they live. In other words, variation in health outcomes, which indicates health inequalities, may be greater in some districts and smaller in others. The application of multilevel modelling is instrumental for handling data that in nested structure (i.e. when data are drawn from different level and outcome is measured at lowest level). Accordingly this study employs a two-level logistic regression model, designed to analyse data aggregated at two distinct levels: with the first level addressing individual factors (like interpersonal trust, and other socio-economic factors) and the second level corresponding to district data (income inequality). MLM is advantageous because it allows for the partitioning of variance at individual and regional levels, offering a detailed analysis of the factors influencing health and trust outcomes. This modelling approach helps researchers explore variations in outcome variables both within and between clusters, identifying the influence of individual-level and broader contextual factors on the outcome variables [26].

The initial model, called the null model, lacks predictors but features a random intercept. This intercept gauges how much the individual variability across districts contributes to odds in dependent variables.

The null model's main goal is calculating the Intra-class correlation (ICC). This measures how much dependent variable variance traces back to district grouping. An ICC above zero suggests intra district outcome similarities, warranting a multi-level model.

1 $$\text{logit}\left({\pi }_{\text{ij}}\right)=\alpha +{u}_{j}$$

where πij represents the probability of an event ( P(Yi = 1)) for individual i in district *j*, *α* is the predicted value of the outcome variable when *x* = 0 (i.e. the intercept) District level effect is measured by the random intercept *α*_*j*_(*j….J*) a linear combination of a grand mean (*α*) and a deviation of (*u*_*j*_). *u*_*j*_ is normally distributed

$${u}_{j}\sim N\left(0, {\sigma }_{u}^{2}\right)$$

$$ICC=\frac{\text{var}\left({u}_{0j}\right)}{\text{var}\left({u}_{0j}\right)+\left({\pi }^{2}/3\right)}$$

var(*u*0*j*) is the random intercept variance. A higher value indicates higher variation between districts;

$$(\pi 2/3)\approx 3.29$$

is the value standard logistic distribution.

In subsequent models, both individual-level (L1) predictors and district-level (L2) predictors are included:

$$\text{logit}(\pi\text{ij})={\alpha }+\text{ uj }+\beta{\text{xij}}$$

Here, βxij represents the coefficient values for the individual-level predictor xij. The terms α and β are the fixed effects, while (uj) represents the district-level random effect.

$$\text{logit}(\pi\text{ij})={\alpha }+{\gamma zj}+\text{uj}+\beta\text{xij}$$

In addition to individual-level predictors, this model includes a district-level fixed effect γzj, where zj represents the district-level attribute.

$$\text{logit}(\pi\text{ij})={\alpha }+{\gamma zj}+\text{uj}+\beta\text{xij}+\theta\text{jxij}$$

This model further introduces an interaction term (θjxij) between the district-level attribute zj and the individual-level predictor xij.

### Marginal effect

In non-linear models, interpreting the significance and implications of interaction terms can be complex when focusing solely on the coefficients' magnitude and direction. To make this interpretation more accessible, I calculated the marginal effects of the interaction terms using the Stata 14 margin command. Marginal effects quantify the change in the predicted probability or expected value of the dependent variable resulting from a one-unit change in an independent variable, holding other variables constant. To further enhance the comprehension of the interaction effect, I plotted the marginal effects, enabling us to visualize the relationships between predictors and the outcome variable, taking into account their interactions.

## Results

Descriptive statistics (Table 1) shows that around 39% population has good SRH and 16% reported to have depressive symptoms. More than 83% respondents has reported to have particularised trust whereas only 56% reported to have generalised trust.

**Table 1:** Descriptive statistics for the variables used in the study

Variables	Description	Mean/ Percentage (weighted)	SD	N	Range /Categories
Health indicators	SRH	38.96		11,227	0= bad health 1=good health
	Mental health (Depression)	15.44		11,230	0= not depressed 1=depressed
Trust	Generalised trust	55.56		11,218	0= no trust = yes
	Particularised trust	83.14		11,191	0= no trust = yes
SES and demographics	Age	50.07	16.6	11,230	18-106
	Male	42		11,230	0= female 1=male
	Urban	26.98		11,230	0=rural 1=urban
	Married	79.04		11,230	0= never married 1=married
	High school and above	18.95		11,229	0= less than high school 1=high school and above
	High income	47.08		11,159	0= low income 1= high income
	Scheduled caste	23.32		11,160	0= non-scheduled caste 1= scheduled caste
	Hindu	83.85		11,230	0= non Hindu 1= Hindu
	Muslim	12.30		11,230	0=non-Muslim 1= Muslim
District level factor	Gini index	0.28	0.06	11,225	0.17-0.47

The estimates by multilevel modelling for good SRH are shown in Table 2. Model 1 is null and consists of only a constant term (β = -0.41, *p* = 0.000) with a district-level random parameter, which accounts for the variation in good self-rated health across districts (district level variance 0.18).

**Table 2:** Mixed effect logistic regression models showing beta coefficient of SRH (good SRH = 1) with generalised trust, particularised trust, micro-level and macro-level predictors, and interaction effects

	SRH	SRH	SRH	SRH	SRH	SRH
	Model 1	Model 2	Model 3	Model 4	Model 5	Model 6
Individual/ micro level factors (level 1)						
Generalized trust (yes = 1)		0.06 (0.04)	0.09* (0.05)	0.09* (0.05)	-0.45* (0.25)	0.29 (0.42)
Particularized trust (yes = 1)		0.36*** (0.06)	0.21*** (0.06)	0.23*** (0.06)	-0.38 (0.34)	-0.07 (0.50)
Age			-0.05*** (0.00)	-0.05*** (0.00)	-0.05*** (0.00)	-0.05*** (0.00)
Male (female = 0)			0.48*** (0.05)	0.48*** (0.05)	0.56** (0.17)	0.40*** (0.12)
Scheduled caste (non-scheduled caste = 0)			-0.05 (0.06)	-0.05 (0.06)	-0.25 (0.19)	-0.1 (0.13)
Hindu (yes1)			0.01 (0.13)	0.01 (0.13)	0.02 (0.13)	0.01 (0.13)
Muslim(yes = 1)			-0.34 (0.15)	-0.35* (0.15)	-0.33* (0.15)	-0.34* (0.15)
Currently married (yes = 1)			-0.05 (0.06)	-0.05 (0.06)	-0.05 (0.06)	-0.05 (0.06)
High school and above (less than high school = 0)			0.51*** (0.06)	0.51*** (0.06)	0.51*** (0.06)	0.51*** (0.06)
High income (low income = 0)			0.21*** (0.05)	0.22*** (0.05)	0.22*** (0.05)	0.22*** (0.05)
Urban(rural = 0)			0.23*** (0.07)	0.23*** (0.07)	0.23*** (0.07)	0.24*** (0.07)
Generalized trust*male					-0.18* (0.09)	-0.17* (0.09)
Generalized trust*scheduled caste					0.13 (0.1)	0.11 (0.11)
Particularized trust*male					0.24* (0.13)	0.23* (0.13)
Particularized trust*scheduled caste					0 (0.14)	0 (0.14)
District/ macro level factors (Level2)						
Gini				0.17 (0.84)		-2.02* (1.23)
micro*macro interaction						
Generalised trust*gini						2.71*** (0.71)
Particularised trust*gini						0.85 (1.03)
Intercept	-0.41*** (0.04)	-0.79*** (0.08)	0.97*** (0.29)	2.12*** (0.46)	2.28*** (0.51)	2.18*** (0.61)
Dist level variance	0.18	0.18	0.25	0.25	0.25	0.24
ICC	0.05	0.05	0.07	0.07	0.07	0.07
N	11,222	11,184	11,042	11,042	11,042	11,042

^*^p < 0.05, **p < 0.01, ***p < 0.001 Standard Error in parentheses

The ICC for model 1 denotes that 5% of the variability in individuals' SRH is due to district-level. differences. In model 2, generalised trust does not significantly impact individuals' good SRH; particularised trust has shown a significant positive impact (β = 0.36, *p* = 0.000) on good SRH.

Model 3 incorporates all individual levels, including trust variables. The association with particularised trust remains significant after adding individuals' socio-economic demographic characteristics; nonetheless, the strength of association drops from β = 0.36 to β = 0.21.

**Fig. 1:**
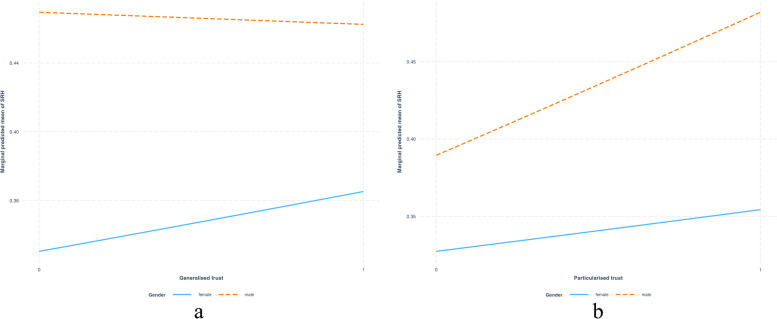
Interaction effect between (**a**) generalised trust & gender on SRH (**b**) particularised trust & gender on SRH

**Fig. 2:**
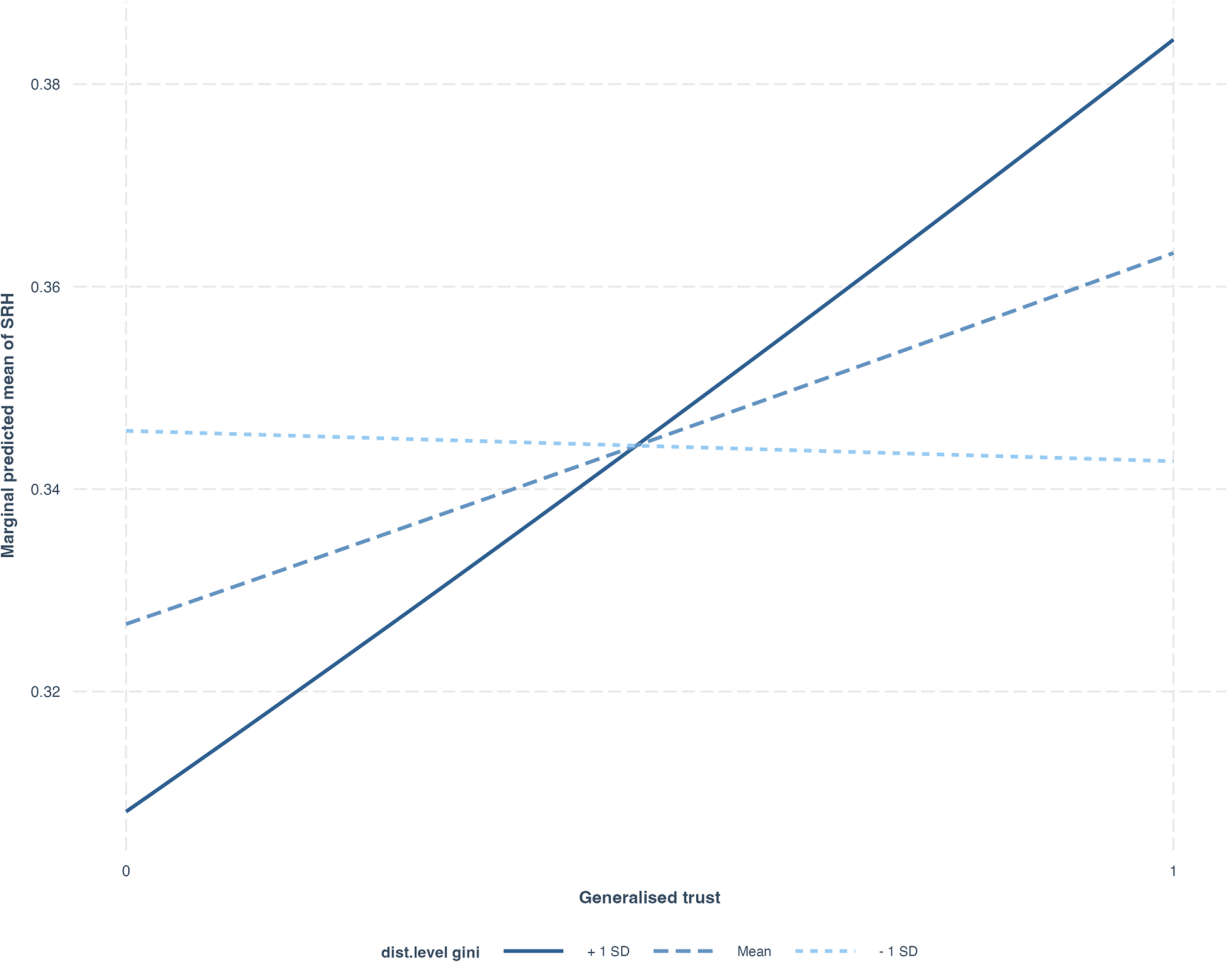
Interaction effect between district level Gini index and generalised trust on SRH

In Model 4, after controlling for other individual and district-level predictors, the beta coefficient for generalised trust is 0.09 with a significance level (*p* < 0.05). This model additionally reveals a substantial increase in the district-level variance (from 0.18 to 0.25), implying that the SRH of individuals has strong contextual (district-level) components that reflect even after controlling individual-level and district-level income inequality. District-level Gini index does not show any association with good SRH after controlling individual-level trust and other individual-level factors. Model 5 incorporated interaction effect (Fig. 1) of social statuses (gender and caste), with generalised and particularised trust. The interactive effect of generalised trust with gender shows a significant negative effect (β = -0.18, *p* < 0.05) on good SRH for males compared to females. The interaction effect suggests that the health benefit of generalised trust is more pronounced for females than males (Fig. 1a). The interactive effect of particularised trust on gender shows that compared to women, men will have good SRH (Fig. 1b). Model 6 incorporated the interaction effect of district-level income inequality (Gini index) with individual level interpersonal trust. The interaction effect between Gini and generalised trust on SRH generalised trustor will have a higher benefit of good SRH in the context of high-income inequality rather than low-income inequality. Figure 2 showing interaction effect between district level Gini index and generalised trust, demonstrates a notable spike in good SRH among highly generalised trustors versus non-trustors when income inequality (Gini) is at its peak (+ 1 SD), as opposed to moderate or low Gini levels.

**Fig. 3:**
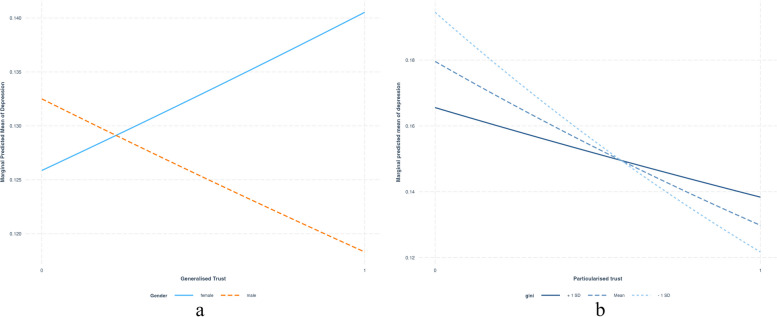
Interaction effect between (**a**) generalised trust and gender on depression (**b**) district level Gini index and particularised trust on depression

Table 3 provides the estimates for depression by multilevel modelling. Model 1 is null and consists of only a constant term (β = -1.77, *p* = 0.000) with a district-level random parameter, which accounts for the variation in having depression across districts (district-level variance 0.69). The ICC for model 1 denotes that 17% of the variability in individuals’ depression is due to district-level differences. In model 2, generalised trust doesn’t show any significant impact on having depression; particularised trust has shown a significant negative impact (β = -0.39 *p* = 0.000) on having depression. In Model 3, social statuses like gender and caste don’t have any significant direct influence on depression. In Model 4, district-level Gini index does not exhibit any link with depression, even after accounting for individual-level trust and other factors. In Model 5, the interaction between generalised trust and gender indicates a significant negative impact (β = -0.26, *p* < 0.05) on male depression compared to females. In simpler terms, this interaction highlights that men are less likely to experience depression than women in the context of higher generalised trust. Figure 3 illustrates a notable decline in the predicted likelihood of depression for males among generalised trustors versus non-trustors, while for women, the likelihood of depression is higher among trustors (Fig. 3a). Model 6 shows that when income inequality is high, the effectiveness of particularised trust in reducing depression diminishes compared to situations of low-income inequality. This trend is reflected in Fig. (3b), where having particularised trust is beneficial for depression reduction across varying income inequality levels. However, when the Gini index of inequality is -1 SD, trustors of particularised trust experience a significant decrease in depression levels.

**Table 3:** Mixed effect logistic regression models showing beta coefficient of depression (having depression = 1) with generalised trust, particularised trust, micro-level and macro-level predictors, and interaction effects

	Depression	Depression	Depression	Depression	Depression	Depression
	Model 1	Model 2	Model 3	Model 4	Model 5	Model 6
Individual/ micro level factors (level 1)						
Generalized trust (yes = 1)		0.05 (0.06)	0.02 (0.06)	0.01 (0.06)	0.56* (0.32)	-0.3 (0.52)
Particularized trust (yes = 1)		-0.39*** (0.07)	-0.28*** (0.07)	-0.27*** (0.07)	-1.10* (0.43)	-0.59 (0.59)
Age			0.03*** (0.00)	0.03*** (0.00)	0.03*** (0.00)	0.03*** (0.00)
Male (female = 0)			-0.09 (0.06)	-0.09 (0.06)	0.24 (0.21)	-0.07 (0.14)
Scheduled caste (non-scheduled caste = 0)			-0.04 (0.07)	-0.04 (0.07)	-0.25 (0.15)	-0.28* (0.16)
Hindu (yes1)			0.22 (0.19)	0.22 (0.19)	0.22 (0.19)	0.24 (0.19)
Muslim(yes = 1)			0.45* (0.21)	0.45* (0.21)	0.45* (0.21)	0.47* (0.21)
Currently married (yes1)			-0.07 (0.07)	-0.07 (0.07)	-0.07 (0.07)	-0.07 (0.07)
High school and above (less than high school = 0)			-0.35*** (0.10)	-0.35*** (0.10)	-0.35*** (0.10)	-0.35*** (0.10)
High income (low income = 0)			-0.33*** (0.06)	-0.32*** (0.06)	-0.33*** (0.06)	-0.32*** (0.06)
Urban(rural = 0)			-0.22* (0.09)	-0.22* (0.09)	-0.22* (0.09)	-0.22* (0.09)
Generalized trust*male					-0.26* (0.12)	-0.27* (0.12)
Generalized trust*scheduled caste					0.27 (0.16)	0.07 (0.14)
Particularized trust*male					0.11 (0.15)	0.14 (0.15)
Particularized trust*scheduled caste					0.25 (0.17)	0.25 (0.17)
District/ macro level factors (Level2)						
Gini				0.73 (1.39)		-1.13 (1.77)
micro*macro interaction						
Generalised trust*Gini						-0.69 (0.97)
Particularised trust *Gini						2.67* (1.31)
Intercept	-1.77*** (0.08)	-1.53*** (0.12)	-3.18*** (0.45)	-3.30*** (0.74)	-3.37*** (0.70)	-2.81*** (0.86)
Dist level variance	0.69	0.69	0.76	0.74	0.76	0.75
ICC	0.17	0.17	0.19	0.18	0.19	0.18
N	11,225	11,185	11,043	11,043	11,043	11,043

^*^p < 0.05, **p < 0.01, ***p < 0.001 Standard Error in parentheses

**Fig. 4:**
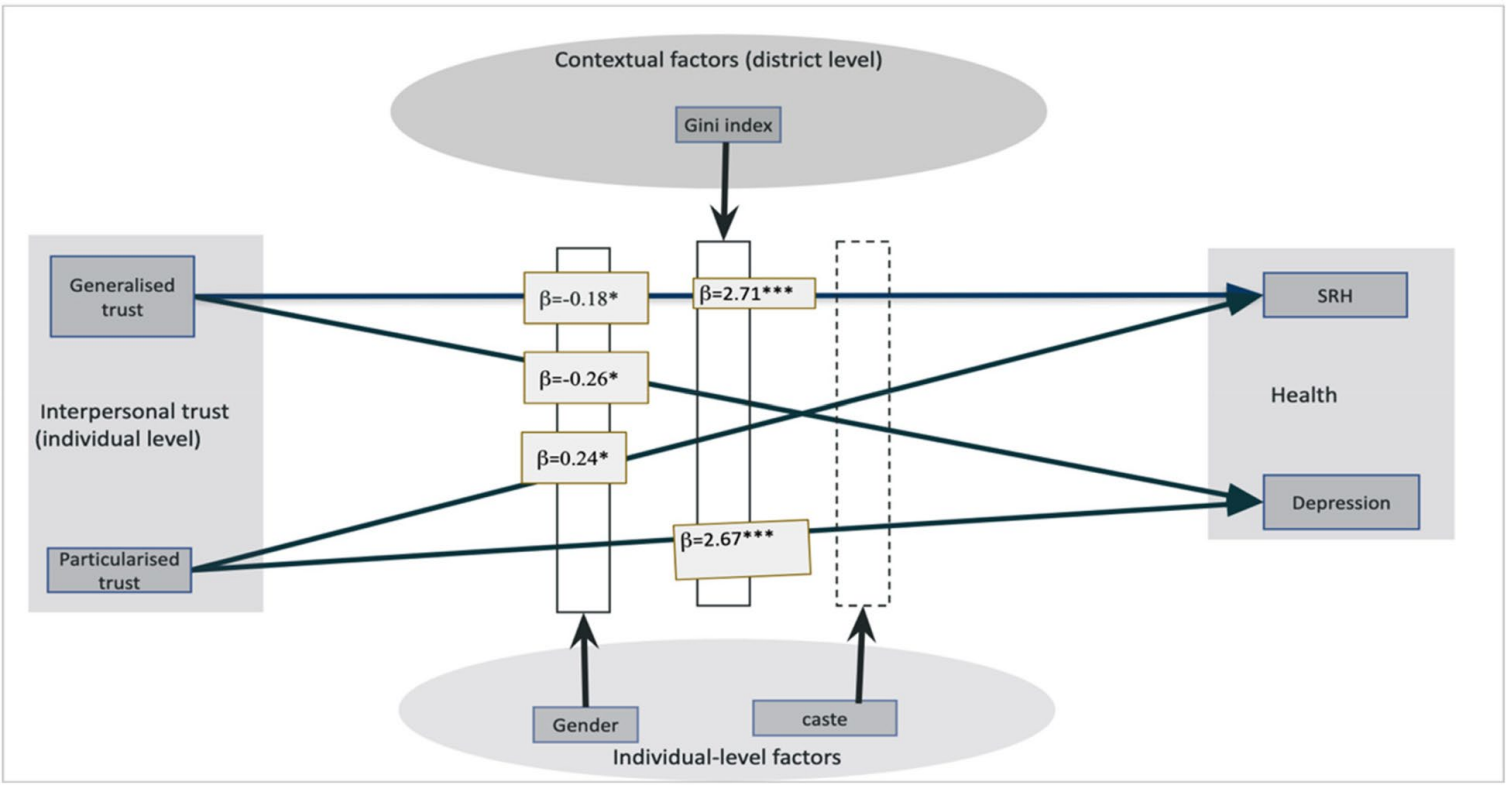
Depicting the interactive effects of individual level and contextual level factors on trust-health relationships. The β values indicate significant interactive effects

The graph (Fig. 4) shows that trust (both generalized and particularized) interacts with gender and economic conditions to differently affect health outcomes in men. Caste does not seem to play a significant role as a moderator in trust-health relationships. However, gender does exert a noteworthy moderating impact on the association between generalised trust and depression and on both generalised and particularised trust with SRH. While generalized trust has a protective effect against depression, particularized trust boosts SRH. However, the benefits of particularized trust are potentially offset by increased risks of depression in contexts of high economic inequality.

## Discussion

Overall, the results from the multilevel regression analysis showed that the association between interpersonal trust and health is mixed and depends on the type of trust (generalised and particularised) in India. Surprisingly, generalised trust does not exhibit a significant direct association with SRH or depression. In contrast, particularised trust demonstrates a health-protective effect on both SRH and depression. These findings address two important gaps in existing research: firstly, they underscore the distinct impacts of generalised and particularised trust on the health status of Indian adults, and secondly, they highlight the heightened relevance of particularised trust as a predictor for SRH and depression, surpassing the influence of generalised trust.

Although limited research exists on the differences between these two forms of trust and their health implications, two cross-national studies using World Values Survey (WVS) data across more than 50 countries have shown the protective effect of particularised trust on SRH, surpassing generalised trust [25, 37]. Further, particularised trust has a more robust connection in developing countries than in developed countries, specially for mental illness. Research from Colombia, Ghana, and South Africa reveals that depression is more positively associated with particularised trust, which focuses on close personal connections like family and friends, rather than generalised trust [2, 3, 28]. This pattern is attributed to the underdeveloped mental health infrastructure in these regions, including challenges such as the integration of mental health care into primary care and a lack of trained professionals [21]. The stigmatisation of mental health issues further diminishes the efficacy of generalised trust in accessing health services, thereby limiting help-seeking behaviours to immediate social circles. In low- and middle-income countries (LMICs), where formal health services are often scarce, the role of family and friends becomes crucial. These informal caregivers provide essential, unpaid support and are pivotal in managing health care, especially for mental health problems [32, 51].

Thus, in contexts like India, where formal support structures are limited and cultural norms emphasise close-knit social networks, particularised trust acts as a more effective protective factor against poor health and depression. Unlike generalised trust, which may help mitigate stress, particularised trust offers direct emotional support and practical help, compensating for the lack of accessible mental health services.

The gender-based moderation effect on the trust-health relationship further indicates that men derive a greater health advantage from having particularised trust to enhance SRH and generalised trust to alleviate depression. On the other hand, women benefit more (than men) from particularised trust concerning SRH improvement. Gender disparities in the health impacts of interpersonal trust can be linked to distinct roles men and women play in trusting connections. Women typically possess higher levels of particularised trust due to their active participation in close social circles. Despite similar social ties, notable gender differences in health outcomes persist. Interestingly, women often serve as essential "nodes" in these networks, offering vital support to other members [17]. Conversely, men's networks are broader but less intense, often relying heavily on their spouses for primary support. Notably, gender differences extend to trust use in marital relationships, consistently highlighting men's greater benefits from these bonds. Men's health gains from marriage often result from their spouses' active promotion of healthy habits and emotional backing. In contrast, women receive less care and support from husbands, leaning more on external confidants [60, 64, 65].

The findings further validate that generalised trust positively impacts men's mental health more than women's. This discrepancy is partly due to women encountering difficulties in forming social networks beyond their immediate circles, leading to lower levels of accessible generalised trust. Since men tend to have more ready access to generalised trust, they naturally enjoy more substantial benefits [4]. The interaction between generalised trust and gender favours women, especially concerning SRH. This implies that even though women might initially have limited access to generalised trust, once established, its impact on health becomes more prominent for them compared to men. In essence, women tend to derive stronger health benefits from generalised trust compared to men.

The cross-level interaction among individual level trust and district-level gini index reveals some interesting findings. Specifically, when generalised trust interacts with income inequality, there is a significant positive effect on good SRH. In essence, while generalised trust might not directly lead to a health-protective outcome in terms of SRH (Table 2), individuals who possess generalised trust can reap benefits if they reside in areas marked by high-income inequality. One way to interpret this finding is that within neighbourhoods or districts marked by pronounced inequality, social capital—particularly in the form of generalised trust—functions as a compensatory mechanism. This mechanism helps counterbalance infrastructural limitations and contributes to enhancing overall health. Prior literature suggests that generalised trust mitigates the negative impact of income inequality on health, particularly in the absence of sufficient government services, as individuals rely on interpersonal trust to navigate insecurity and vulnerability [31].

While particularised trust has demonstrated a significant health protecting impact on depression, its interaction with district-level Gini suggests a contrasting outcome – a positive effect on having depression. Prior research has explored regions with high income inequality; economically disadvantaged communities often find themselves confined within tight-knit, insular social networks. Such close-knit, impoverished communities possess a wealth of particularised trust that aids them in coping, but they lack the resources of expansive bridging networks necessary for advancement [70].While having particularised trust or a reliable support system can enhance health by offering informal assistance, it can also contribute to mental distress, particularly among caregivers. In a nutshell, particularised trust, especially concerning socioeconomic disadvantage, can have health-damaging consequences, as observed in some studies [22, 46]. This phenomenon highlights the complex relationship between particularised trust, socioeconomic disadvantage, and its potential health consequences. Notably, the interactive effect of district level Gini index on generalised and particularised trust appear to be in line with previous studies showing the risks of relying too much on particularised trustees or bonding social networks may have a detrimental effect on mental health specially in district where, inequality is high [22]. Further it confirms the protective effect of generalised trust or diverse social networks for SRH as they may help buffer against the detrimental influences of neighbourhood income inequality [19]. The buffering effect of generalised trust in high-income inequality districts indicates that communities that rely on networks to fulfil various needs can harness the power of these networks to mitigate poor health.

## Limitation and future direction

The paper presents several limitations that offer directions for future research. Firstly, using cross-sectional data limits the ability to establish causal inferences, making it challenging to examine the dynamics of trust and health over time. Thus, a natural extension of this paper is using longitudinal analysis for the simultaneous monitoring of changes in trust levels and health statuses. It is important to acknowledge the possibility of reverse causality or bidirectional relationships when examining the moderation effects of income inequality on the trust-health relationship. This approach is particularly essential for testing hypotheses such as H3a and H3b, which propose that income inequality moderates the relationship between interpersonal trust and health outcomes (SRH and depression). Poor health significantly reduces an individual's productivity, increases absenteeism, and raises the likelihood of unemployment, leading to a widening income gap between healthy and unhealthy individuals. A chronic health condition contributes to substantial medical expenses, putting households at risk of poverty, especially in areas where comprehensive health insurance policies don't exist. Future research should consider methodologies that can account for and test these potential bidirectional effects. To test the robustness of the findings against potential reverse causality, it would be beneficial to emphasize the importance of employing longitudinal and cross-lagged panel designs in future research.

Secondly, the study focuses on individual identities such as gender and caste but does not incorporate the concept of intersectionality. Employing intersectionality theory in future research could provide deeper insights into how interpersonal trust perpetuates entrenched inequalities across multiple social identities and affects health outcomes. Future research should consider employing this framework to examine the nuanced effects of compounded social factors on health outcomes, providing deeper insights into the dynamic interplay of trust and health within diverse social contexts.

Thirdly, the validation of self-rated health measures is problematic due to their subjective nature. This subjectivity can lead to reporting heterogeneity and may cause significant over- or underestimations of health inequalities. Future studies should consider including objective health measures alongside subjective assessments, such as clinical assessments or biomarkers, to evaluate health outcomes better.

Finally, the generalizability of the findings is limited, as the data is drawn from only six states in India, each with unique geographical and socio-economic characteristics. Future research should aim to replicate the study across a broader range of geographic and demographic settings to enhance the generalizability of the findings and verify the limit of the broader applicability of the results in different contexts.

## Conclusion

While a considerable body of research in India has primarily focused on systemic challenges like inadequate healthcare infrastructure, imbalanced resource distribution, and socioeconomic disparities, this study notably breaks new ground by highlighting the importance of investigating psychosocial resources as potential determinants of health.

The findings reveal that while generalized trust does not directly affect health outcomes, particularized trust shows a strong protective effect on both self-rated health (SRH) and mental well-being, indicated by depression. Additionally, the interactive effects of trust and social context suggest that in high-income inequality districts, generalised trust can buffer against the detrimental effects of inequality, whereas reliance on particularised trust may exacerbate mental health issues.

For India, these findings imply that enhancing interpersonal trust, particularly generalised trust, could be a pivotal strategy for improving health outcomes. Policymakers should focus on strengthening social security measures, local institutions, and community safety to foster trust. Community-based programs and neighbourhood resources like schools and clubs can also play a crucial role in building social trust. On an international scale, these insights can inform global health policies by demonstrating the importance of psychosocial factors in health interventions. Countries with similar socio-economic challenges could benefit from strategies aimed at enhancing social trust to improve health outcomes. Effective allocation of development aid should consider interventions that bolster community networks and trust.

Future research should explore the mechanisms through which interpersonal trust impacts health in various contexts and examine innovative ways to leverage social networks for health improvement in resource-limited settings. Strengthening social trust at both individual and community levels is essential for achieving better health outcomes and addressing health inequalities globally.

## Supplementary Information

Supplementary Material 1.

## Data Availability

The study utilizes a secondary data which is available on formal request from the World Health Organization Multi-Country Studies Data Archive through its online platform (http://apps.who.int/healthinfo/systems/surveydata/index.php/catalog) for researchers who meet the criteria for access to confidential data.
